# APR-246—The Mutant TP53 Reactivator—Increases the Effectiveness of Berberine and Modified Berberines to Inhibit the Proliferation of Pancreatic Cancer Cells

**DOI:** 10.3390/biom12020276

**Published:** 2022-02-08

**Authors:** James Andrew McCubrey, Stephen L. Abrams, Linda S. Steelman, Lucio Cocco, Stefano Ratti, Alberto M. Martelli, Paolo Lombardi, Agnieszka Gizak, Przemysław Duda

**Affiliations:** 1 Department of Microbiology and Immunology, Brody School of Medicine, East Carolina University, Greenville, NC 27858, USA; abramss@ecu.edu (S.L.A.); lssteelman@gmail.com (L.S.S.); 2 Department of Biomedical and Neuromotor Sciences, Università di Bologna, 40126 Bologna, Italy; lucio.cocco@unibo.it (L.C.); stefano.ratti@unibo.it (S.R.); alberto.martelli@unibo.it (A.M.M.); 3 Naxospharma, Via Giuseppe Di Vittorio 70, 20026 Novate Milanese, Italy; p.lombardi@naxospharma.eu; 4 Department of Molecular Physiology and Neurobiology, University of Wrocław, 50-335 Wroclaw, Poland; agnieszka.gizak@uwr.edu.pl (A.G.); przemyslaw.duda2@uwr.edu.pl (P.D.)

**Keywords:** TP53, PDAC, berberine, NAX compounds, mutant TP53 reactivators

## Abstract

Pancreatic ductal adenocarcinoma (PDAC) is the most common form of pancreatic cancer. In ~75% of PDAC, the tumor suppressor *TP53* gene is mutated. Novel approaches to treat cancer involve compounds called mutant TP53 reactivators. They interact with mutant TP53 proteins and restore some of their growth suppressive properties, but they may also interact with other proteins, e.g., TP63 and TP73. We examined the ability of the TP53 reactivator APR-246 to interact with eleven modified berberine compounds (NAX compounds) in the presence and absence of WT-TP53 in two PDAC cell lines: the MIA-PaCa-2, which has gain of function (GOF) TP53 mutations on both alleles, and PANC-28, which lacks expression of the WT TP53 protein. Our results indicate the TP53 reactivator-induced increase in therapeutic potential of many modified berberines.

## 1. Introduction

Pancreatic cancer is the second leading cause of death [1,2]. Most pancreatic cancers are pancreatic ductal adenocarcinomas (PDAC) [1,2,3]. Their treatment consists of surgical resection of the affected part of the pancreas. Unfortunately, the tumor usually comes back, often due to metastasis to other organs [4,5], and once PDAC has metastasized, it is difficult, if not impossible, to successfully treat. For decades, chemotherapy has been used as treatment modality for various cancer patients [5,6] and PDAC patients. However, it is usually a palliative and not curative approach [3,6,7,8].

Over the past 25–40 years, many of the genes implicated in PDAC have been identified [8,9,10,11,12,13]. Perhaps two of the best-known mutated genes are the oncogene *KRAS* and the tumor suppressor gene *TP53* [8,9,10,11,12,13].

Numerous types of the *TP53* gene mutations have been documented in PDAC and other cancers [14,15,16,17,18,19,20,21]. Some mutations are deletions or frame shift mutations and may result in loss of either all or parts of the TP53 protein (truncations) [19]. These genetic events could result in TP53 null cells. Such cells could also result from epigenetic alterations, which suppress the expression of the *TP53* gene.

Point mutations at the *TP53* gene may result in a protein with an altered activity in comparison to the WT protein. In some cases, these mutant TP53 proteins may have altered growth regulatory properties and diverse biochemical effects. Such mutations are referred to as GOF (gain of function) mutations [14,15,16,17,18]. The mutant GOF TP53 proteins may or may not induce the transcription of TP53-regulated genes or may induce the transcription of other genes, which are not normally targets of TP53. In addition, the GOF mutations may change interactions of the TP53 proteins with other regulatory proteins [14,15,16,17,18]. For example, a mutant TP53 can in some cases interact with oncogenic KRas [22,23,24].

Despite our knowledge of key genes involved in PDAC, therapy remains difficult, and PDAC patients usually have a poor outcome. Thus, there is dire need to develop more effective approaches to treat PDAC.

Identification of low molecular weight TP53 activators (reactivators) that interact with the mutant TP53 protein and restore some of its activity provide an approach to suppress the effects of GOF TP53 mutations [25,26,27]. APR-246, also known as PRIMA-1MET and Eprenetapopt, is a compound with such properties, and it has been examined in several clinical trials [28,29,30]. APR-246 (2-hydroxymethyl-2-methoxymethyl-3-quinuclidinone) is a prodrug and it is converted into 2-methylene-3-quinuclidinone (MQ) to become the active form, which is a Michael acceptor [31].

The cytotoxic effects of APR-246 have been studied in various cancer types, e.g., hematopoietic and prostate cancers [32,33]. In addition, the abilities of APR-246 to suppress proliferation in breast cancer [34,35], colorectal cancer (CRC) [36], glioblastoma [37], head and neck cancers [38], melanoma [39], ovarian cancer [40,41], PDAC [42,43], and small and non-small cell lung cancer cells have been demonstrated [44,45]. In addition, APR-246 has been shown to overcome the drug resistance of certain primary ovarian cancer cells [41].

Part of the effects of APR-246 may be through the generation of reactive oxygen species (ROS), which could alter the structure of the mutant TP53 protein [38,46], but APR-246 can also bind the critical cysteine residues in the core binding domain of mutant TP53 protein and change its conformation [47]. This can result in reactivation of TP53 activity [25,26,44]. Mutant TP53 “reactivators” may also bind TP63 and TP73 proteins [48]. These TP53 “reactivators” stabilize the proteins and maintain their correctly folded conformation.

BBR is an isoquinoline quaternary alkaloid (a 5,6-dihydrodibenzo[a,g]quinolizinium) derivative [49,50,51,52], which is present in numerous plants. It has been consumed as a nutraceutical for many aliments for centuries. More recently, BBR has been shown to improve cancer therapy, as it suppresses many genes associated with cell growth, inflammation, prevention of apoptosis, invasion, and metastasis [49,50]. Treatment of “normal cells” with BBR appears to have minimal effects. In contrast, BBR suppressed the proliferation of cancerous (e.g., breast, colon, liver, pancreatic) cells [51,52,53,54,55], inhibited the growth of MIA-PaCa-2 PDAC cells in a xenograft nu/nu mouse model [56], and did not affect the weight of the mice and appeared to be well tolerated [56]. 

BBR appears to act on cells through multiple mechanisms. For example, BBR treatment can result in the production of reactive oxygen species (ROS) [49,52], induction of autophagy, apoptosis, and cellular senescence, suppression of events associated with migration and metastasis such as inhibition of cytokine/chemokine expression, (interleukin-6 (IL-6), tumor necrosis factor-α, monocyte chemo-attractant protein 1 (MCP1) and COX-2 production [49,57,58,59,60]. BBR treatment induces AMP-activated protein kinase (AMPK) [61] and TP53 activation via ROS and inhibits mTORC1 phosphorylation [56]. BBR can also induce double strand DNA breaks (DBS) [62]. In some cases, these processes are TP53 dependent.

**Figure 1:**
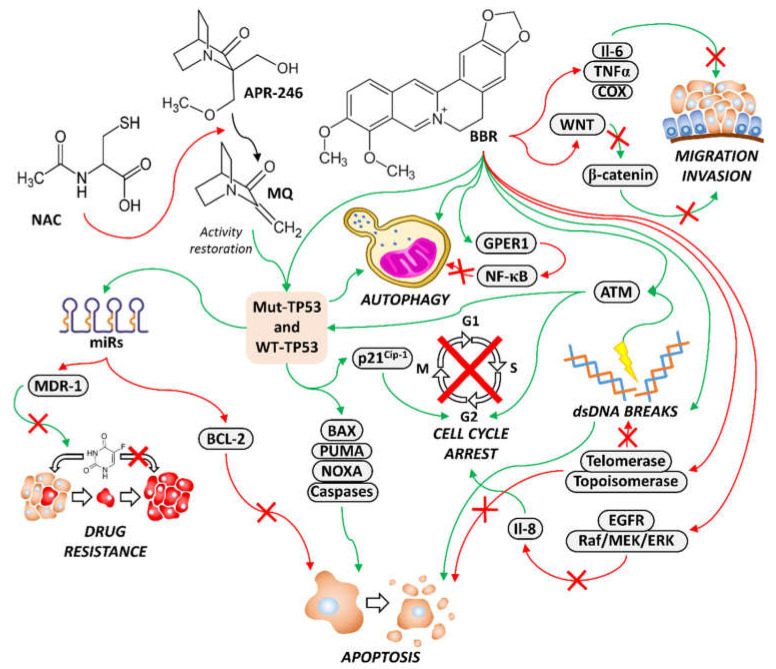
Diagram representing effects of APR-246, BBR and NAX compounds on TP53 activity and cell growth and apoptosis. Restoration of some of the activity of WT TP53 will have effects on expression of miRs, cell cycle regulators and apoptotic molecules. NAC, N-acetylcysteine; APR-246, 2-hydroxymethyl-2-methoxymethyl-3-quinuclidinone; MQ, 2-methylene-3-quinuclidinone; BBR, berberine; Mut-TP53, mutant TP53; green arrows, promote activity; red arrows, block activity; red X in arrow, activity is blocked.

A diagram of the effects of APR-246 and BBR on various aspects of cancer growth is presented in Figure 1.

As expected for a nutraceutical, BBR normally is not highly toxic or growth inhibitory. Thus, multiple approaches have been made to increase the effectiveness of BBR to suppress cell growth and other processes. A panel of modified BBRs has been developed (NAX compounds) [63,64,65]. Some of these modified BBRs interact with their intracellular targets more effectively than BBR [66,67,68,69,70]. For example, NAX014 inhibited β-catenin signaling at 100-fold lower concentrations than BBR [70].

In the following studies, we examined the abilities of the mutant TP53 reactivator APR-246 to increase the abilities of BBR and NAX compounds to inhibit the proliferation of two PDAC cell lines, one of which has GOF mutations in the p53 protein and the other of which is TP53 null, before and after introduction of WT-TP53.

## 2. Materials and Methods

### 2.1. Cell Lines

The sources of the PDAC cell lines and culture conditions have been extensively described previously [71,72,73,74,75,76,77,78,79].

### 2.2. Sources of APR-246, BBR and NAX Compounds

The sources of these compounds have been previously described. APR-246 was bought from Selleck Chemicals (Houston, TX, USA). BBR was purchased from MilliporeSigma (Saint Louis, MO, USA). Dr. Paolo Lombardi provided the NAX compounds (Naxospharma (Novate Milanese, Italy).

### 2.3. Transduction of MIA-PaCa-2 and PANC-28 Cells with a Vector Encoding WT-TP53 or an Empty Vector Control

The sources of vectors encoding WT-TP53 and pLXSN been described previously [80,81]. Retroviral transduction was previously described [75,82,83,84].

### 2.4. Measurements of Cellular Proliferation

Cellular growth assays were performed as described [75,81,82,83,84] by MTT assays. In the experiments where a low dose of APR-246 was added with BBR and the various NAX compound, on day 0, the indicated cell lines were seeded in the 96-well plates. On day 1, various 2-fold dilutions were made of the BBR and NAX compounds in centrifuge tubes, and then they were added in triplicate wells to the 96-well plates in duplicate. After the BBR and NAX compounds were added, 12.5 nM APR-246 was added to one set of plates, while an equal volume of tissue culture medium was added to the other set of plates.

### 2.5. Clonogenicity Assays

**Figure 2:**
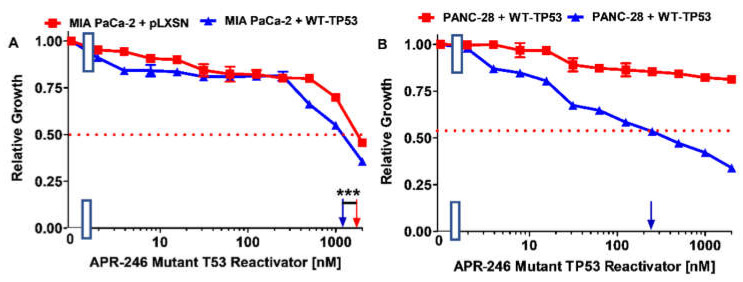
The ability of APR-246 to suppress the growth of PDAC cells is dependent on the presence of either an WT-TP53 or mut-TP53 protein. Panel (**A**) MIA-PaCa-2 + pLXSN cells (control cells lacking WT-TP53 but containing GOF TP53 and pLXSN) (red squares) and MIA-PaCa-2 + WT-TP53 cells (containing both WT-TP53 and GOF TP53) (blue triangles). Panel (**B**) PANC-28 + pLXSN cells (lacking detectable TP53 but containing and pLXSN) (red squares) and PANC-28 + WT-TP53 cells (containing both WT-TP53 and GOF TP53) (blue triangles). These experiments were repeated 4 times, and similar results were obtained. Blue and red arrows on the X-axis indicate when the IC_50s_ were estimated, *** = *p* < 0.0001, NS, not statistically significant.

**Figure 3:**
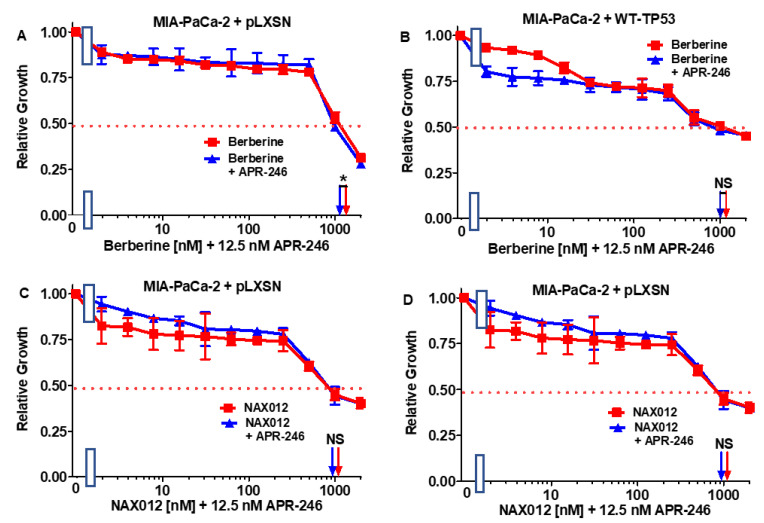
APR-246 does not enhance the responses of berberine or NAX012 to MIA-PaCa-2 cells containing or lacking WT-TP53 protein. Panel (**A**) MIA-PaCa-2 + pLXSN cells treated with different concentrations of BBR (red squares) or with different concentrations of BBR and a low dose of APR-246 (blue triangles). Panel (**B**) MIA-PaCa-2 + WT-TP53 cells treated with different concentration of BBR (red squares) or with different concentrations of BBR and a low dose of APR-246 (blue triangles). Panel (**C**) MIA-PaCa-2 + pLXSN cells treated with different concentrations of NAX012 (red squares) with different concentrations of NAX012 and a low dose of APR-246 (blue triangles). Panel (**D**) MIA-PaCa-2 + WT-TP53 cells treated with different concentration of NAX012 (red squares) or with different concentrations of NAX012 and a low dose of APR-246 (blue triangles). These experiments were repeated 4 times, and similar results were observed. * = *p* < 0.05, NS, not statistically significant.

Clonogenicity assays were carried out as described [75,76,77,78]. Low doses of APR-246 BBR and NAX060 were determined by titration experiments to not suppress growth by more than 50% Figure 2 and Figure 3 [77,78,79].

### 2.6. Statistical Analysis

MTT experiments were set up as described [77,78,79] in triplicate, with a series of 11 serial 2-fold dilutions as well as an untreated control. The means and standard deviation of the samples were calculated by GraphPad Prism software (San Diego, CA, USA). Statistical significance of the comparison of two means of IC_50s_ of two different culture conditions (+/− 12.5 nM APR-246) or clonogenicity of two different cell types was calculated using the GraphPad QuickCalcs software using an unpaired *t* test with a 95% confidence interval.

## 3. Results

### 3.1. Effects of the Mutant TP53 Reactivator on Two PDAC Cell Lines

The effects of the mutant TP53 reactivator were examined on two PDAC cell lines: MIA-PaCa-2, which contains GOF TP53 genes on both alleles [71], and PANC-28, which does not express detectable levels of the TP53 protein [73]. Into these cells, we introduced either a vector containing the WT-TP53 cDNA [79] or the control parental vector (pLXSN) lacking any insert [80]. Both vectors encode resistance to G418.

MIA-PaCa-2 + WT-TP53 cells were about 1.5-fold more sensitive to APR-246 than MIA-PaCa-2 + pLXSN cells (Figure 2A). PANC-28 + pLXSN cells were not sensitive to APR-246 (IC_50_ > 2000 nM). However, upon introduction of WT-TP53 into the cells, they became > 8-fold more sensitive to APR-246 (IC_50_ = 250 nM) (Figure 2B).

### 3.2. Interactions between Berberine (BBR), Chemically Modified BBRs (NAX Compounds) and APR-246 in MIA-PaCa-2 + pLXSN and MIA-PaCa-2 + WT-TP53 Cells

To determine whether the effects of BBR and NAX compounds on the growth of PDAC cells could be enhanced by the mutant TP53 reactivator APR-246, MIA-PaCa-2 cells containing or lacking TP53 were plated in the presence and absence of a low concentration of APR-246. The addition of 12.5 nM APR-246 had a mild effect on the sensitivity of MIA-PaCa-2 + pLXSN and MIA-PaCa-2 + WT-TP53 cells. As in both cases, the IC_50_ of the combination of BBR and APR-246 decreased only 1.1-fold (Figure 3A,B). These results are also summarized in Table 1.

**Figure 4:**
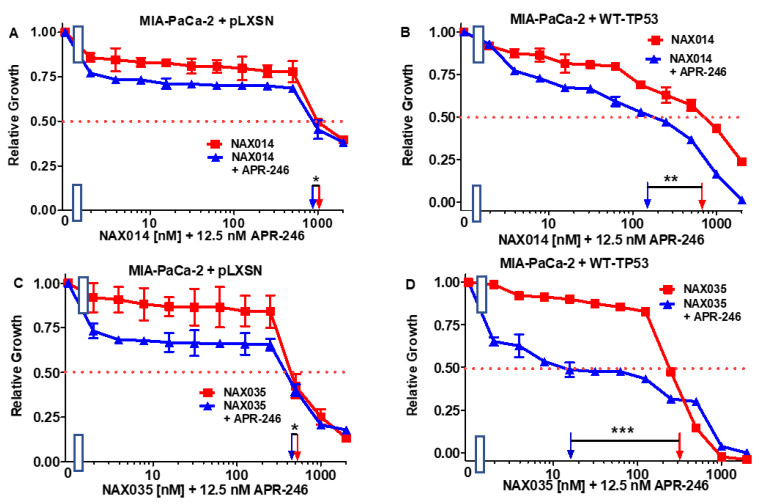
APR-246 is more effective in enhancing the effects of NAX014 and NAX035 in MIA-PaCa-2 cell when WT-TP53 is present. Panel (**A**) MIA-PaCa-2 + pLXSN cells treated with different concentrations of NAX014 (red squares) or with different concentrations of NAX014 and a low dose of APR-246 (blue triangles). Panel (**B**) MIA-PaCa-2 + WT-TP53 cells treated with different concentrations of NAX014 (red squares) or with different concentrations of NAX014 and a low dose of APR-246 (blue triangles). Panel (**C**) MIA-PaCa-2 + pLXSN cells treated with different concentrations of NAX035 (red squares) or with different concentrations of NAX035 and a low dose of APR-246 (blue triangles). Panel (**D**) MIA-PaCa-2 + WT-TP53 cells treated with different concentrations of NAX035 (red squares) or with different concentrations of NAX035 and a low dose of APR-246 (blue triangles). The experiments were repeated 4 times, and similar results were observed. *** = *p* < 0.0001, ** = *p* < 0.005, * = *p* < 0.05, NS, not statistically significant.

Then, the combination of APR-246 with 11 structurally different NAX compounds was examined in MIA-PaCa-2 + WT-TP53 and MIA-PaCa-2 + pLXSN. The low dose of APR-246 did not lower the IC_50_ of NAX012 in either MIA-PaCa-2 + WT-TP53 or MIA-PaCa-2 + pLXSN cells (Figure 3C,D), and had only a mild effect on the sensitivity of MIA-PaCa-2 + pLXSN to NAX014 as the IC_50_ decreased 1.1-fold (Figure 4A). In contrast, the presence of APR-246 had a more significant effect on the sensitivity of MIA-PaCa-2 + WT-TP53 cells to NAX014, as the IC_50_ decreased 4.4-fold from 700 to 160 nM (Figure 4B).

In the presence of APR-246, the sensitivity of MIA-PaCa-2 + pLXSN cells to NAX035 decreased only slightly (1.1-fold), but in MIA-PaCa-2 + WT-TP53 cells, the TP53 reactivator reduced the NAX035 IC_50_ almost 19-fold (Figure 4C,D).

**Figure 5:**
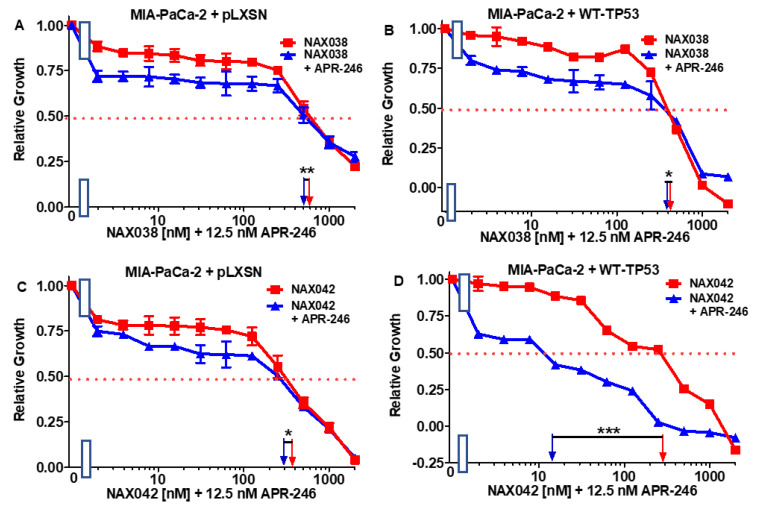
APR-246 has minimal effects on the responses of NAX038 on MIA-PaCa-2 cells in the presence and absence of WT-TP53, while APR-246 enhances the responses of NAX042 in MIA-PaCa-2 cells when WT-TP53 is present. Panel (**A**) MIA-PaCa-2 + pLXSN cells treated with different concentrations of NAX038 (red squares) or with different concentrations of NAX054 and a low dose of APR-246 (blue triangles). Panel (**B**) MIA-PaCa-2 + WT-TP53 cells treated with different concentrations of NAX038 (red squares) or with different concentrations of NAX038 and a low dose of APR-246 (blue triangles). Panel (**C**) MIA-PaCa-2 + pLXSN cells treated with different concentration of NAX042 (red squares) or with different concentrations of NAX042 and a low dose of APR-246 (blue triangles). Panel (**D**) MIA-PaCa-2 + WT-TP53 cells treated with different concentrations of NAX042 (red squares) or with different concentrations of NAX042 and a low dose of APR-246 (blue triangles). The measurements were repeated 4 times, and similar results were observed. *** = *p* < 0.0001, ** = *p* < 0.005, * = *p* < 0.05.

Addition of the low dose of APR-246 had a mild effect on the sensitivity of both MIA-PaCa-2 + pLXSN MIA-PaCa-2 + WT-TP53 cells to NAX038 as the IC_50_ decreased, respectively, 1.2- and 1.3-fold (Figure 5A,B). 

A dose of APR-246 had a similar mild effect on the sensitivity of MIA-PaCa-2 + pLXSN to NAX042 (Figure 5, Panel C). In contrast, APR-246 had a more significant effect on the sensitivity of MIA-PaCa-2 + WT-TP53 cells to NAX042 as the IC_50_ decreased 23-fold, from 300 to 13 nM (Figure 5D).

**Figure 6:**
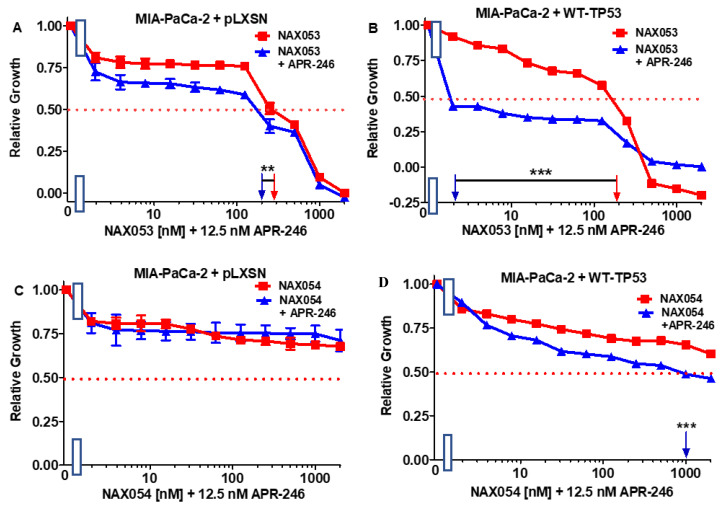
APR-246 enhances the responses of NAX053 in MIA-PaCa-2 when WT-TP53 is present, while APR-236 has less effects on the responses of NAX054 on MIA-PaCa-2 cells in the presence of WT-TP53. Panel (**A**) MIA-PaCa-2 + pLXSN cells treated with different concentrations of NAX053 (red squares) or with different concentrations of NAX053 and a low dose of APR-246 (blue triangles). Panel (**B**) MIA-PaCa-2 + WT-TP53 cells treated with different concentrations of NAX053 (red squares) or with different concentrations of NAX053 and a low dose of APR-246 (blue triangles). Panel (**C**) MIA-PaCa-2 + pLXSN cells treated with different concentration of NAX054 (red squares) or with different concentrations of NAX054 and a low dose of APR-246 (blue triangles). Panel (**D**) MI-PaCa-2 + WT-TP53 cells treated with different concentrations of NAX054 (red squares) or with different concentrations of NAX054 and a low dose of APR-246 (blue triangles). The measurements were repeated 4 times, and similar results were observed. *** = *p* < 0.0001, ** = *p* < 0.005.

APR-246 decreased the sensitivity of MIA-PaCa-2 + pLXSN cells to NAX053 only 1.5-fold (Figure 6, Panel A), but it had a more significant on the sensitivity of MIA-PaCa-2 + WT-TP53 cells to NAX053 as the IC_50_ decreased 100-fold from 200 to 2 nM (Figure 6B).

Previously we have observed that NAX054 did not have any significant effects on MIA-PaCa-2 cells [77,78]. Likewise, NAX054 did not have effects on MIA-PaCa-2 + pLXSN in the presence of ABR-246. However, in MIA-PaCa-2 + WT-TP53 cells, APR-246 reduced the IC_50_ to NAX054 at least two-fold (Figure 6C,D).

**Figure 7:**
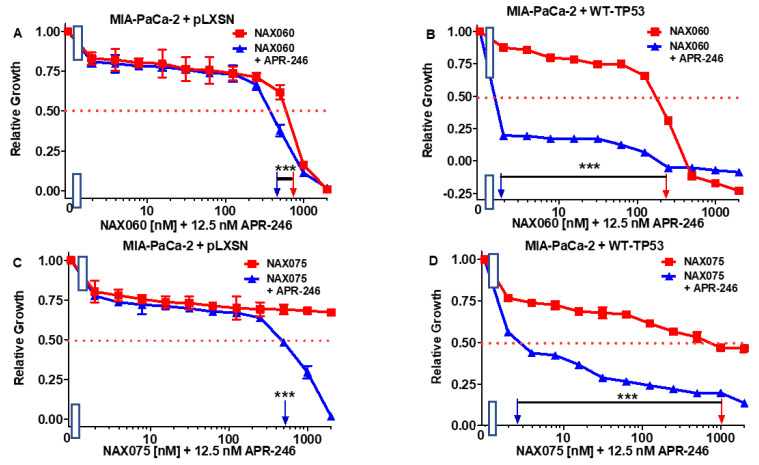
APR-246 is more effective in enhancing the effects of NAX060 and NAX075 in MIA-PaCa-2 cell when WT-TP53 is present. Panel (**A**) MIA-PaCa-2 + pLXSN cells treated with different concentrations of NAX060 (red squares) or with different concentrations of NAX060 and a low dose of APR-246 (blue triangles). Panel (**B**) MIA-PaCa-2 + WT-TP53 cells treated with different concentrations of NAX060 (red squares) or with different concentrations of NAX060 and a low dose of APR-246 (blue triangles). Panel (**C**) MIA-PaCa-2 + pLXSN cells treated with different concentration of NAX075 (red squares) or with different concentrations of NAX075 and a low dose of APR-246 (blue triangles). Panel (**D**) MI-PaCa-2 + WT-TP53 cells treated with different concentrations of NAX075 (red squares) or with different concentrations of NAX075 and a low dose of APR-246 (blue triangles). The measurements were repeated 4 times, and similar results were observed. *** = *p* < 0.0001.

The low dose of APR-246 had an effect on the sensitivity of MIA-PaCa-2 + pLXSN cells to NAX060 as the IC_50_ decreased two-fold from 800 to 400 nM (Figure 7, Panel A), but it had a more significant effect on the sensitivity of MIA-PaCa-2 + WT-TP53 cells to NAX060 as the IC_50_ decreased 110-fold from 220 to 2 nM (Figure 7B).

The low dose of APR-246 had an effect on the sensitivity of MIA-PaCa-2 + pLXSN cells to NAX075 as the IC_50_ decreased >4-fold from >2000 to 500 nM (Figure 7C), but it had a more significant effect on the sensitivity of MIA-PaCa-2 + WT-TP53 cells to NAX075 as the IC_50_ decreased 357-fold from 1000 to 2.8 nM (Figure 7D).

**Figure 8:**
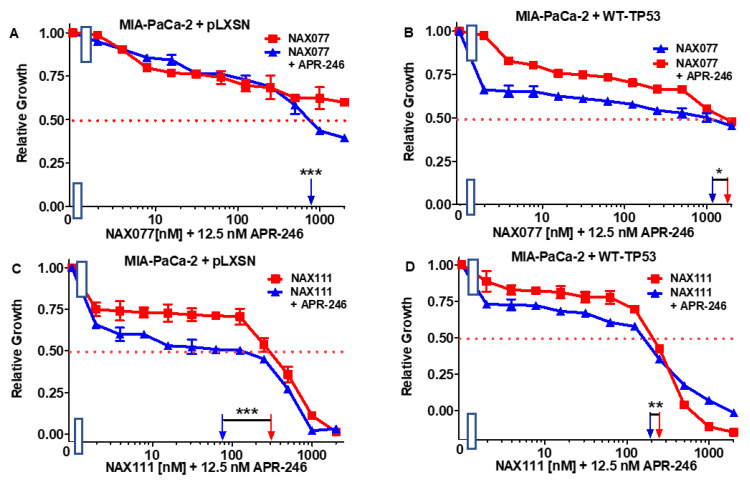
APR-246 has minimal effects on enhancing the effects of NAX077 and NAX111 in the presence and absence of WT-TP53. Panel (**A**) MIA-PaCa-2 + pLXSN cells treated with different concentrations of NAX075 (red squares) or with different concentrations of NAX075 and a low dose of APR-246 (blue triangles). Panel (**B**) MIA-PaCa-2 + WT-TP53 cells treated with different concentrations of NAX075 (red squares) or with different concentrations of NAX075 and a low dose of APR-246 (blue triangles). Panel (**C**) MIA-PaCa-2 + pLXSN cells treated with different concentration of NAX111 (red squares) or with different concentrations of NAX111 and a low dose of APR-246 (blue triangles). Panel (**D**) MI-PaCa-2 + WT-TP53 cells treated with different concentrations of NAX111 (red squares) or with different concentrations of NAX111 and a low dose of APR-246 (blue triangles). The measurements were repeated 4 times, and similar results were observed. *** = *p* < 0.0001, ** = *p* < 0.005, and * = *p* < 0.05.

APR-246 reduced >2.5-fold the IC_50_ of NAX077 in the MIA-PaCa-2 + pLXSN but only 1.7-fold in MIA-PaCa-2 + WT-TP53 (Figure 8A,B).

Similarly, APR-246 had a stronger effect on the sensitivity of MIA-PaCa-2 + pLXSN than of MIA-PaCa-2 + WT-TP53 cells to NAX111, as the IC_50_ for this compound decreased three-fold in the former cells and only 1.3-fold in the latter cells (Figure 8C,D).

In summary, the addition of a low dose of APR-246 could increase the cytotoxic effects of certain NAX compounds on MIA-PaCa-2 cells. The most significant effect—over 100-fold increase in sensitivity to NAX—was observed with MIA-PaCa-2 + WT-TP53 with NAX060.

Surprisingly, APR-246 has stronger effects on MIA-PaCa-2 + pLXSN than of MIA-PaCa-2 + WT-TP53 when combined with NAX077 and NAX111.

### 3.3. Abilities of Low Doses of BBR or NAX060 to Increase the Cytotoxicity of APR-246 and Decrease Clonogenicity of MIA-PaCa-2 Cells Containing and Lacking WT-TP53

**Figure 9:**
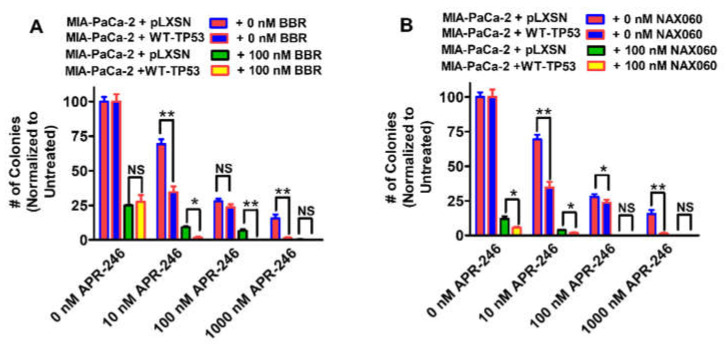
Both berberine and NAX60 can increase the effects of APR-246 on clonogenicity of MIA-PaCa-2 cells in the presence and absence of WT-TP53. The effects of ABR-246 on colony formation in the presence of low doses of BBR (Panel (**A**)) or NAX060 (Panel (**B**)) were examined. MIA-PaCa-2 + pLXSN in the absence of BBR or NAX060 (red bars), MIA-PaCa-2 + WT-TP53 in the absence of BBR or NAX060 (blue bars), MIA-PaCa-2 + pLXSN in the presence of APR-246 and 100 nM BBR (green bars), MIA-PaCa-2 + WT-TP53 in the presence of APR-246 of 100 nM NAX060 (Panel (**B**)) (yellow bars). The clonogenicities for each cell type and each treatment condition were repeated 3 times, and similar results were observed. ** = *p* < 0.005, * = *p* < 0.05, NS, not statistically significant.

In subsequent experiments, we tested a promising combination: APR-246, and NAX060, and APR-246 with BBR as the benchmark, in terms of their abilities to influence the clonogenicity of the MIA-PaCa-2 cells containing and lacking WT-TP53. This time, we used different concentrations of APR-246 and low doses of BBR or NAX060 (Figure 9).

APR-246 inhibited the clonogenicity of MIA-PaCa-2 + pLXSN and MIA-PaCa-2 + WT-TP53 cells in a dose-dependent fashion. Furthermore, the addition of a low dose of berberine resulted in increased suppression of growth (Figure 9A). The effects of APR-246 were greater when the MIA-PaCa-2 + WT-TP53 cells were treated with higher concentrations of APR-246 and BBR than in MIA-PaCa-2 + pLXSN cells. Higher APR-246 concentrations had stronger effects on MIA-PaCa-2 + WT-TP53 than control cells which lacked WT TP53.

Likewise, a low dose of NAX060 suppressed clonogenicity in both MIA-PaCa-2 + pLXSN and MIA-PaCa-2 + WT-TP53 cells (Figure 9B). As observed previously (Figure 3A,B and Figure 7A,B), NAX060 suppressed growth to a greater extent than BBR, and a higher level of growth inhibition was observed in MIA-PaCa-2 + WT-TP53 than in MIA-PaCa-2 + WT-TP53 cells.

**Figure 10:**
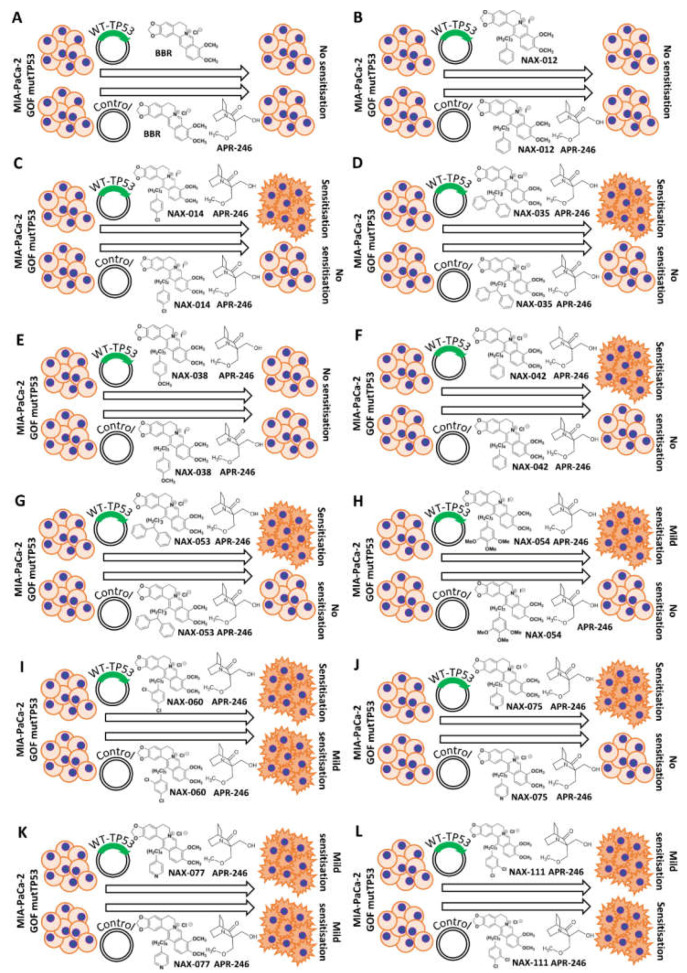
Chemical structures of BBR and NAX compounds and the potential effects of the mutant TP53 reactivator APR-246 to increase their toxicity of MIA-PaCa-2 + pLXSN and MIA-PaCa-2 + WT-TP53 cells. Abilities of APR-246 to interact with BBR and NAX compounds and to cause increased growth inhibition in MIA-PaCa-2 cells either in the presence of GOF m-TP53 or GOF-mTP53 + introduced WT-TP53. Mut-TP53, mutant TP53. (**A**) = Berberine, (**B**) = NAX012, (**C**) = NAX014, (**D**) = NAX035, (**E**) = NAX038, (**F**) = NAX042, (**G**) = NAX053, (**H**) = NAX054, (**I**) = NAX060, (**J**) = NAX075, (**K**) = NAX077, (**L**) = NAX111.

A summary of the effects of combination of APR-246, BBR an NAX compounds in both MIA-PaCa-2 + pLXSN and MIA-PaCa-2 + WT-TP53 cells is presented in Figure 10. Included in this figure as well are the structures of BBR and the NAX compounds.

### 3.4. Interactions between Berberine (BBR), Chemically Modified BBRs (NAX Compounds) and APR-246 in PANC-28 + pLXSN and PANC-28 + WT-TP53 Cells

Next, we determined whether the effects of BBR and NAX compounds on the growth of PDAC cells could be enhanced by the low concentration (12.5 nM) of mutant TP53 reactivator APR-246 also in PANC-28 cells containing or lacking WT TP53.

**Figure 11:**
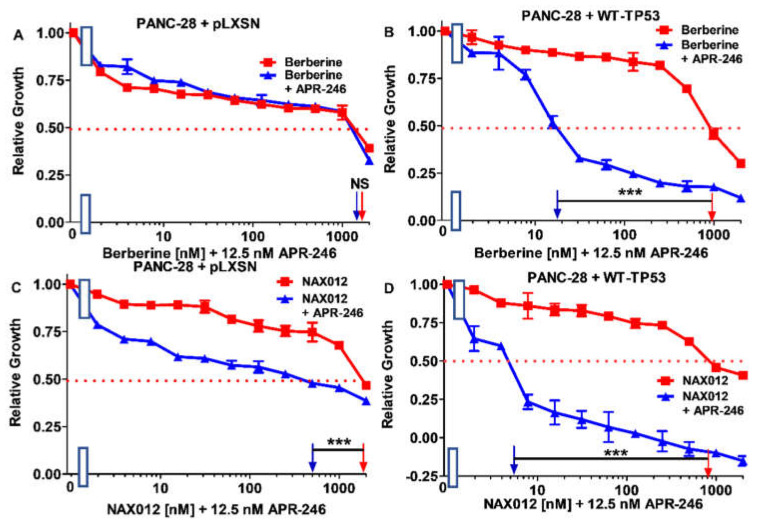
APR-246 enhances the effects of berberine when WT-TP53 is present in PANC-28 cells, while APR-246 enhances the effects of NAX012 in PANC-28 cells in the presence of WT-TP53 greater than in the absence of WT-TP53. Panel (**A**) PANC-28 + pLXSN cells treated with different concentrations of BBR (red squares) or PANC-28 + pLXSN cells treated with different concentrations of BBR and a low dose of APR-246 (blue triangles). Panel (**B**) PANC-28 + WT-TP53 cells treated with different concentration of BBR (red squares) or with different concentrations of BBR and a low dose of APR-246 (blue triangles). Panel (**C**) PANC-28 + pLXSN cells treated with different concentrations of NAX012 (red squares) or with different concentrations of NAX012 and a low dose of APR-246 (blue triangles). Panel (**D**) PANC-28 + WT-TP53 cells treated with different concentration of NAX012 (red squares) or with different concentrations of NAX012 and a low dose of APR-246 (blue triangles). These experiments were repeated 3 times and similar results were observed. *** = *p* < 0.0001, and NS, not statistically significant.

Addition of APR-246 had a mild effect on the sensitivity of PANC + pLXSN as the IC_50_ on the combination of BBR and APR-246 decreased only 1.1-fold, from 1600 to 1500 nM (Figure 11A). In contrast, when PANC-28 + WT-TP53 cells were treated with APR-246, the IC_50_ for BBR decreased 55.6-fold from 1000 to 18 nM (Figure 11B). These results are summarized in Table 2.

**Figure 12:**
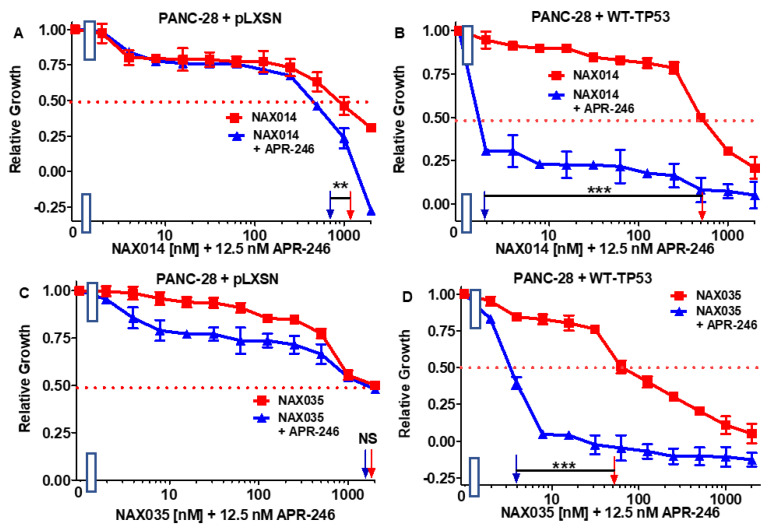
APR-246 enhances the effects of NAX014 greater when WT-TP53 is present in PANC-28 cells, while APR-246 enhances the effects of NAX035 in PANC-28 cells in the presence of WT-TP53 but not in the absence of WT-TP53. Panel (**A**) PANC-28 + pLXSN cells treated with different concentrations of NAX014 (red squares) or with different concentrations of NAX014 and a low dose of APR-246 (blue triangles). Panel (**B**) PANC-28 + WT-TP53 cells treated with different concentrations of NAX014 (red squares) or with different concentrations of NAX014 and a low dose of APR-246 (blue triangles). Panel (**C**) PANC-28 + pLXSN cells treated with different concentrations of NAX035 (red squares) or with different concentrations of NAX035 and a low dose of APR-246 (blue triangles). Panel (**D**) PANC-28 + WT-TP53 cells treated with different concentrations of NAX035 (red squares) or with different concentrations of NAX035 and a low dose of APR-246 (blue triangles). The measurements were repeated 3 times, and similar results were observed. *** = *p* < 0.0001, ** = *p* < 0.005, NS, not statistically significant.

**Figure 13:**
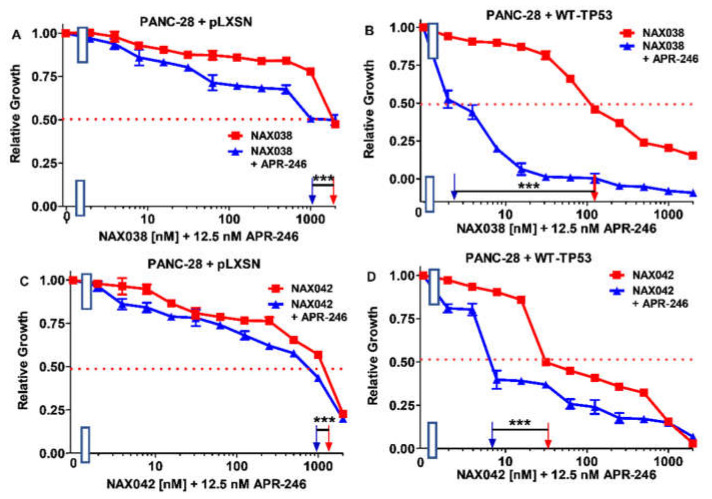
APR-246 enhances the effects of NAX038 and NAX043 greater when WT-TP53 is present in PANC-28 cells than when WT-TP53 is absent. Panel (**A**) PANC-28 + pLXSN cells treated with different concentrations of NAX038 (red squares) or with different concentrations of NAX038 and a low dose of APR-246 (blue triangles). Panel (**B**) PANC-28 + WT-TP53 cells treated with different concentrations of NAX038 (red squares) or with different concentrations of NAX038 and a low dose of APR-246 (blue triangles). Panel (**C**) PANC-28 + pLXSN cells treated with different concentration of NAX042 (red squares) or with different concentrations of NAX042 and a low dose of APR-246 (blue triangles). Panel (**D**) PANC-28 + WT-TP53 cells treated with different concentrations of NAX042 (red squares) or with different concentrations of NAX042 and a low dose of APR-246 (blue triangles). The measurements were repeated 3 times, and similar results were observed. *** = *p* < 0.0001.

**Figure 14:**
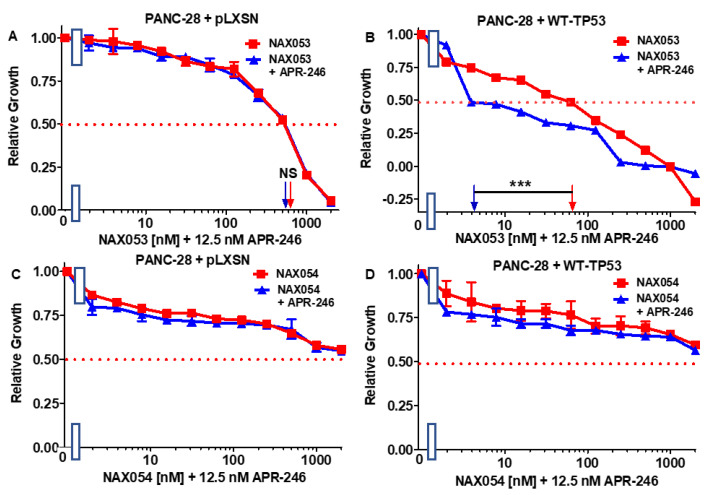
APR-246 enhances the effects of NAX053 when WT-TP53 is precent in PANC-28 cells but not in the absence of WT-TP53, while APR-246 does not increase the effects of NAX054 in the presence or absence of WT-TP53. Panel (**A**) PANC-28 + pLXSN cells treated with different concentrations of NAX053 (red squares) or with different concentrations of NAX053 and a low dose of APR-246 (blue triangles). Panel (**B**) PANC-28 + WT-TP53 cells treated with different concentrations of NAX053 (red squares) or with different concentrations of NAX053 and a low dose of APR-246 (blue triangles). Panel (**C**) + PANC-28 + pLXSN cells treated with different concentration of NAX054 (red squares) or with different concentrations of NAX054 and a low dose of APR-246 (blue triangles). Panel (**D**) PANC-28 + WT-TP53 cells treated with different concentrations of NAX054 (red squares) or with different concentrations of NAX054 and a low dose of APR-246 (blue triangles). The measurements were repeated 3 times, and similar results were observed. *** = *p* < 0.0001, NS, not statistically significant.

**Figure 15:**
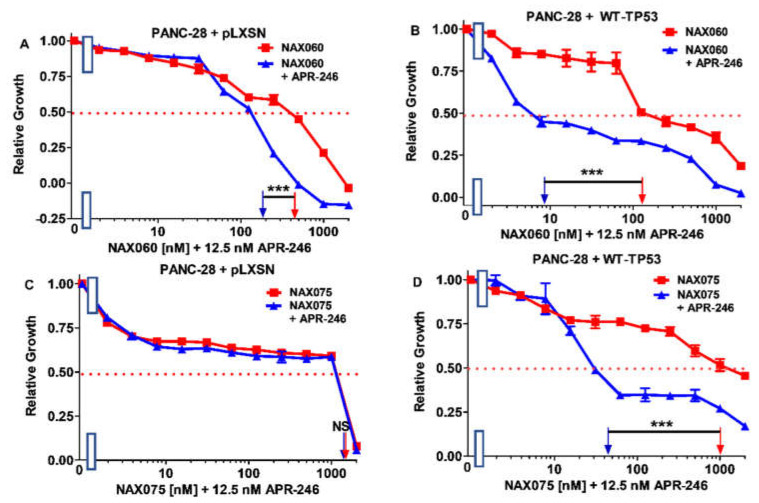
APR-246 enhances the effects of NAX060 on PANC-28 cells more in the presence of WT-TP53 than in the absence of WT-TP53, while APR-246 enhances the effects of NAX075 in the presence of WT-TP53 but not in the absence of WT-TP53. Panel (**A**) PANC-28 + pLXSN cells treated with different concentrations of NAX060 (red squares) or with different concentrations of NAX060 and a low dose of APR-246 (blue triangles). Panel (**B**) PANC-28 + WT-TP53 cells treated with different concentrations of NAX060 (red squares) or with different concentrations of NAX060 and a low dose of APR-246 (blue triangles). Panel (**C**) PANC-28 + pLXSN cells treated with different concentration of NAX075 (red squares) or with different concentrations of NAX075 and a low dose of APR-246 (blue triangles). Panel (**D**) PANC-28 + WT-TP53 cells treated with different concentrations of NAX075 (red squares) or with different concentrations of NAX075 and a low dose of APR-246 (blue triangles). The measurements were repeated, and similar results were observed. *** = *p* < 0.0001, NS, not statistically significant.

**Figure 16:**
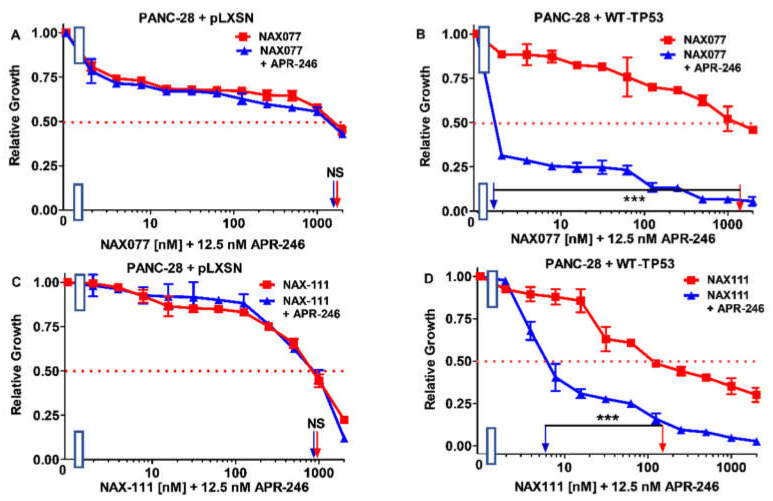
APR-246 enhances the effects of NAX077 and NAX11 on PANC-28 cells in the presence of WT-TP53 but not in the absence of WT-TP53. Panel (**A**) PANC-28 + pLXSN cells treated with different concentrations of NAX075 (red squares) or with different concentrations of NAX075 and a low dose of APR-246 (blue triangles). Panel (**B**) PANC-28 + WT-TP53 cells treated with different concentrations of NAX075 (red squares) or with different concentrations of NAX075 and a low dose of APR-246 (blue triangles). Panel (**C**) PANC-28 + pLXSN cells treated with different concentration of NAX111 (red squares) or with different concentrations of NAX111 and a low dose of APR-246 (blue triangles). Panel (**D**) PANC-28 + WT-TP53 cells treated with different concentrations of NAX111 (red squares) or with different concentrations of NAX111 and a low dose of APR-246 (blue triangles). The measurements were repeated 3 times, and similar results were observed. *** = *p* < 0.0001, and NS, not statistically significant.

Then, we tested the same panel of compounds as in the case of MIA-PaCa-2 cells on PANC-28 cells. The addition of APR-246 had no or only moderate effects on the sensitivity of PANC-28 + pLXSN to NAX compounds (Figure 11, Figure 12, Figure 13, Figure 14, Figure 15 and Figure 16). The best results for PANC-28 + pLXSN cells were obtained in the combination with NAX012: APR-246 lowered the IC_50_ for this compound four-fold (Figure 11C).

In turn, in the case of PANC-28 + WT-TP53 cells, the low dose of APR-246 was more effective in reducing the IC_50_ for almost all the tested NAX compounds (Figure 11, Figure 12, Figure 13, Figure 14, Figure 15 and Figure 16). The only exception was NAX054, whose IC_50_ remained unchanged by APR-246 (Figure 14D). This is, however, in agreement with our previous studies showing that NAX054 did not have any significant effects on PANC-28 cells [81].

The most impressive influence of the low dose of APR-246 was observed in the case of NAX077 as the IC_50_ for this compound in PANC-28 + WT-TP53 cells decreased 1000-fold from 1500 to 1.5 nM (Figure 16B). All data are summarized in Table 2.

### 3.5. Abilities of Low Doses of BBR or NAX060 to Increase the Cytotoxicity of APR-246 and Decrease Clonogenicity of PANC-28 Cells Containing and Lacking WT-TP53

**Figure 17:**
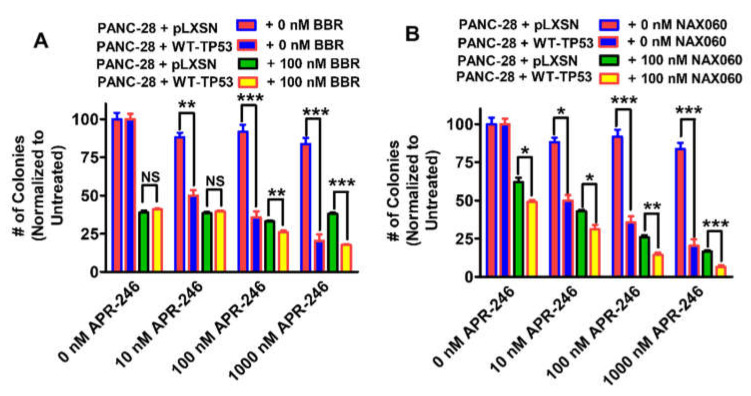
Both berberine and NAX60 can increase the effects of APR-246 on clonogenicity of PANC-28 cells in the presence WT-TP53, but in the absences of WT-TP53 they have less effects on clonogenicity. The effects of ABR-246 on clonogenicity in the presence of low doses of BBR (Panel (**A**)) or NAX060 (Panel (**B**)) were examined. PANC-28 + pLXSN in the absence of BBR or NAX060 (red bars), PANC-28 + WT-TP53 in the absence of BBR or NAX060 (blue bars), PANC-28 + pLXSN in the presence of APR-246 and 100 nM BBR (green bars), MIA-PaCa-2 + WT-TP53 in the presence of APR-246 and100 nM NAX060 (Panel (**B**)) (yellow bars). The colony formation abilities were repeated 3 times for each cell type and each treatment condition and similar results were observed. *** = *p* < 0.0001, ** *p* < 0.005, * *p* < 0.05 and NS = not statistically significant.

We compared the effectiveness of low doses of NAX060 and BBR to influence the clonogenicity of the PANC-28 cells containing and lacking WT-TP53 in the presence of increasing concentrations of APR-246 (Figure 17).

APR-246 inhibited the colony formation of PANC-28 + WT-TP53 cells in a dose-dependent fashion. In contrast, APR-246 had less effects on PANC-28 + pLXSN cells.

Furthermore, the addition of a low dose of BBR resulted in increased suppression of growth in PANC-28 + pLXSN cells (Figure 17A). However, the addition of increasing concentrations of APR-246 did not result in a significant decrease in clonogenicity in PANC-28 + pLXSN cells. The effects of APR-246 were greater when the PANC-28 + WT-TP53 cells were treated with higher concentrations of APR-246 and BBR than in PANC-28 + pLXSN cells.

In contrast, NAX060 had some effects on PANC-28 + pLXSN cells that were greater at higher doses of APR-246. Likewise, a low dose of NAX060 suppressed clonogenicity in both PANC-28 + pLXSN and PANC-28 + WT-TP53 cells (Figure 17B). As observed previously (Figure 15C), NAX060 suppressed growth to a greater extent than BBR (Figure 17A), and a higher level of growth inhibition was observed in PANC-28 + WT-TP53 cells than in PANC-28 + pLXSN cells.

In summary, PANC-28 + pLXSN cells were more resistant to certain NAX compounds (e.g., NAX035, NAX053, NAX060) than to others. PANC-28 + WT-TP53 cells were more sensitive to NAX compounds such as NAX035, NAX038, NAX042, NAX053, NAX060, NAX077, and NAX111 than to others. APR-246 could sensitize PANC-28 + pLXSN cells to certain NAX compounds such as NAX014 and NAX060. However, the effects of APR-246 when combined with BBR and many NAX compounds such as NAX012, NAX014, NAX035, NAX038, NAX042, NAX053, NAX060, NAX075, NAX077 and NAX111 were much more significant in PANC-28 + WT-TP53 cells.

## 4. Discussion

APR-246 is a drug that has been clinically evaluated in patients with certain types of cancer. Berberine (BBR) is a nutraceutical that has been examined for many types of health problems including cancer. Modified BBRs have been developed that have enhanced effects in certain pre-clinical cancer models. Some studies have indicated that some effects of BBR are TP53 dependent [69]. The possible effects of combining the mutant TP53 reactivator APR-246 and NAX compounds on PDAC cells containing and lacking WT TP53 activity have not been previously described.

**Figure 18:**
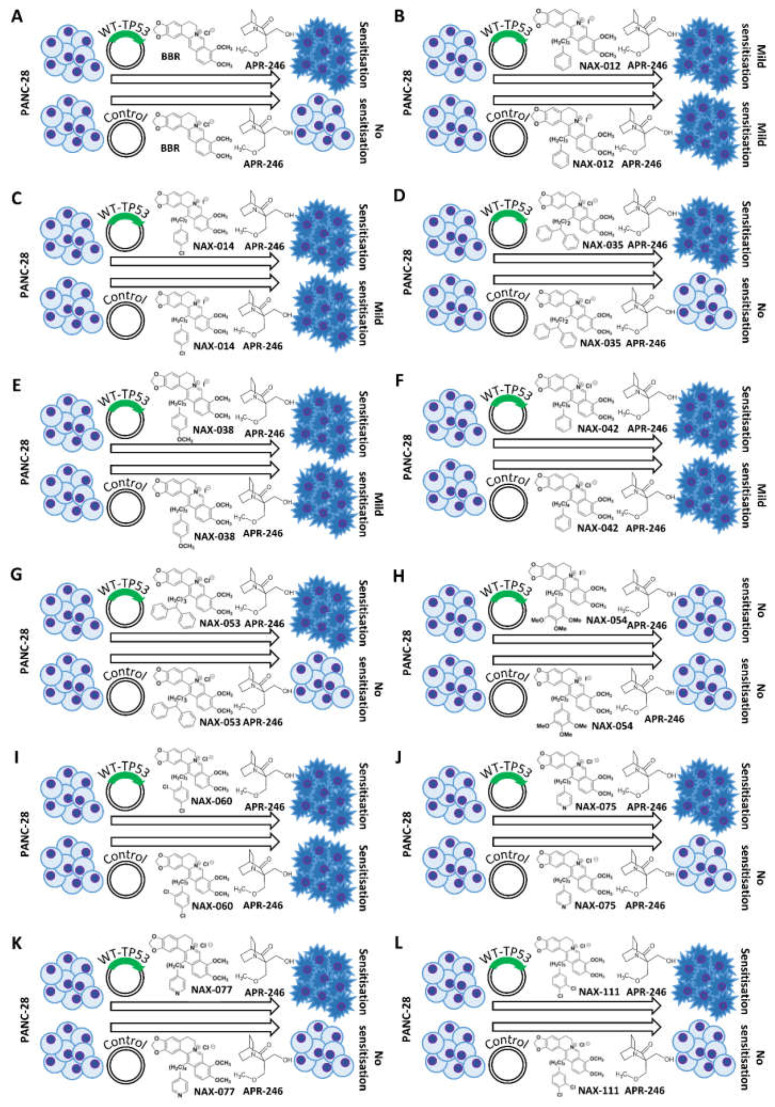
Chemical structures of BBR and NAX compounds and the potential effects of the mutant TP53 reactivator APR-246 to increase their toxicity of PANC-28 + pLXSN and PANC-28 + WT-TP53 cells. Abilities of APR-246 to interact with BBR and NAX compounds and cause increased growth inhibition in PANC-28 cells either in the presence of non-expressed (TP53 null) or the same cells with introduced WT-TP53. (**A**) = Berberine, (**B**) = NAX012, (**C**) = NAX014, (**D**) = NAX035, (**E**) = NAX038, (**F**) = NAX042, (**G**) = NAX053, (**H**) = NAX054, (**I**) = NAX060, (**J**) = NAX075, (**K**) = NAX077, (**L**) = NAX111.

The MIA-PaCa-2 cell line contains GOF mutant TP53. The addition of WT-TP53 increased the sensitivity of MIA-PaCa-2 cells to a low dose of APR-246. BBR showed some cytotoxic effects on MIA-PaCa-2 + pLXSN and MIA-PaCa-2 + WT-TP53. The addition of a low dose of APR-246 did not significantly change the effect of BBR. Similar results were observed with NAX012. A summary of these results is presented in Figure 18.

In contrast, the combination of APR-246 and some of the other NAX compounds (e.g., NAX014, NAX035, NAX042, NAX053, NAX060, NAX075) significantly reduced the viability of MIA-PaCa-2 cells in the presence of WT-TP53.

We have previously observed that certain NAX compounds, namely NAX035, NAX038, NAX042, NAX053, NAX060, and NAX111, are more toxic toward MIA-PaCa-2 cells + pLXSN than BBR [77,78,83]. In contrast, MIA-PaCa-2 cells are less sensitive to NAX012, NAX014, NAX075 and NAX077 than to BBR, and not sensitive to NAX054. Likewise, the addition of WT-TP53 to TP53 null PANC-28 cells to the combination of APR-246 and BBR and most NAX compounds.

The differences in chemical modifications of the BBR backbone structures of the various NAX are presented in Figure 10 and Figure 18. The chemical modifications of the NAX compounds have (un)substituted (hetero) aromatic moieties bonded to position 13 of the parent alkaloid BBR skeleton through a hydrocarbon (C) linker of variable lengths. The structures of the NAX compounds are presented in Figure 10 and Figure 18 and [78,83].

NAX012 and NAX042 have additional phenyl moieties with hydrocarbon (C) linkers of different lengths. NAX012 has a 3 C linker and has been prepared as iodide salt. NAX042 has a 4 C linker and has been prepared as chloride salt. NAX042 was more effective in suppressing proliferation of MIA-PaCa-2 cells than NAX012.

NAX012 and NAX014 suppressed proliferation of MIA-PaCa-2 cells moderately; however, the addition of WT-TP53 resulted in increased sensitivity to the combination of NAX014 and APR-246 substantively, while in the case of NAX012 it has no effect. Clearly, the number of C in the linkers present in the NAX012 or NAX014 compounds and the presence of a chlorine atom on the added monophenyl present in NAX014 can affect their ability to inhibit PDAC growth.

NAX035 and NAX053 have two phenyl moieties (benzhydryl) with C linkers of different lengths. NAX035 has a 2 C linker while NAX035 has a 3 C linker. Both compounds were effective in suppressing the proliferation of MIA-PaCa-2 cells. The addition of a low dose of APR-246 exhibited similar sensitizing effects on treatment of MIA-PaCa-2 + WT-TP53 cells to both NAX035 and NAX053.

NAX075 and NAX077 have additional one heterocycle (pyridine) with C linkers of different lengths. NAX075 has a 3 C linker, while NAX077 has a 4 C linker. These compounds are similar in structure. They had minimal effects on suppressing the proliferation on MIA-PaCa-2 + pLXSN cells. The addition of a low dose of APR-246 had more substantial effects on NAX075 than NAX077 treatment of MIA-PaCa-2 + WT-TP53 cells.

NAX038 and NAX054 have multiple electron-releasing substituents on the introduced monophenyl moiety. NAX038 was effective in suppressing the proliferation of MIA-PaCa-2 + pLXSN and MIA-PaCa-2 + WT-TP53 cells. In contrast, NAX054 with three methoxy (OCH_3_) groups on the added phenyl moiety did not suppress proliferation of either MIA-PaCa-2 + pLXSN or MIA-PaCa-2 + WT-TP53 cells. The addition of a low dose of APR-246 had modest on the effects of NAX038 on either MIA-PaCa-2 + pLXSN or MIA-PaCa-2 + WT-TP53 cells. A low dose of APR-246 was modest on the effects on NAX054 in MIA-PaCa-2 + WT-TP53 cells.

NAX014, NAX060 and NAX111 have multiple electron-withdrawing substituents on the introduced monophenyl moiety. NAX060 and NAX111 had similar effects on MIA-PaCa-2 cells. However, introduction of WT-TP53 increased the effects of addition of a low concentration of APR-246 when added with NAX060, but not NAX111.

In terms of clonogenicity, the effects of APR-246 were increased when WT-TP53 was present in MIA-PaCa-2 + WT-TP53 cells. The effects of BBR by itself were not different in the absence of APR-246. However, the effects of NAX060 were different in the absence of APR-246 in the presence and absence of WT-TP53. As observed by MTT analysis, NAX060 was more potent than BBR in suppressing growth.

These modifications of the BBR core structure had different effects on suppression of MIA-PaCa-2 growth and sensitivity in combination with APR-246. It is possible that the different modifications of the BBR core structure in the NAX compounds affect the ability of the molecules to interact with the promoter regions of key genes involved in regulation of cell growth, induction of apoptosis and other important biochemical processes.

The effects of the combination of APR-246, BBR and NAX compounds were also examined in PANC-28 cells containing and lacking WT-TP53. PANC-28 cells lack detectable TP53 [74]. The APR-246 mutant TP53 reactivator had little effect on PANC-28 cells. In contrast to the results observed with MIA-PaCa-2 cells, introduction of WT-TP53 in PANC-28 cells increased their sensitivity to BBR, NAX012, NAX014, NAX035, NAX038, NAX042, NAX053, NAX060, NAX060, NAX075, NAX077 and NAX111 but not NAX054 in terms of IC_50_ analysis in MTT growth assays.

APR-246 did not have significant effects on colony formation of PANC-28 + pLXSN. However, the addition of WT-TP53 sensitized the PANC-28 + WT-TP53 to APR-246. The colony formation of PANC-28 + pLXSN cells was sensitive to BBR and NAX060. The addition of WT-TP53 sensitized the PANC-28 + WT-TP53 cells more to the combination of APR-246 and NAX060 than to BBR.

In terms of clonogenicity, NAX060 was more effective in suppressing colony formation than BBR. This agrees with what we observed by MTT growth assays. The addition of WT-TP53 increased the sensitivity of PANC-28 cells to APR-246.

Our studies point to the value of knowing what type of TP53 mutation(s) present in cancer patients that may be treated with APR-246 and next-generation-related compounds. These compounds may not have a significant effect on cells that have deleted or silenced TP53 expression. While this observation may be obvious, there are other TP53-related molecules such as TP63 and TP73, which could be stabilized by the APR-246 compound in PANC-28 + pLXSN cells. However, we observed that treatment of PANC-28 + pLXSN was not sensitive to the combination of BBR and APR-246. In contrast, PANC-28 + WT-TP53 were highly sensitive to the combination of APR-246 and BBR. Our studies indicate that APR-246 can interact with certain modified BBRs to increase their cytotoxicity on PDAC cells. These interactions are often increased in the presence of WT TP53.

## Figures and Tables

**Table 1:** Effects of WT-TP53 on the sensitivity of MIA-PaCa-2 PDAC cells to APR-246 ^1^.

Berberine or NAX Compound	MIA-PaCa-2 + pLXSN (-APR-246)	MIA-PaCa-2 + pLXSN (+12.5 nM APR-246)	*p* Value and Statistical Significance Symbol	Fold Change +/− APR-246	MIA-PaCa-2 + WT-TP53 (-APR-246)	MIA-PaCa-2 + WT-TP53 (+12.5 nM APR-246)	*p* Value and Statistical Significance Symbol	Fold Change +/− APR-246
Berberine	1200 nM	1100 nM	*p* = 0.047, *	1.1 × ↓	1100 nM	1000 nM	*p* = 1, NS	1.1 × ↓
NAX012	1000 nM	1000 nM	*p* = 1, NS	1 ×	700 nM	700 nM	*p* = 1, NS	1 ×
NAX014	1100 nM	1000 nM	*p* = 0.048, *	1.1 × ↓	700 nM	160 nM	*p <* 0.0001, ***	4.4 × ↓
NAX035	500 nM	450 nM	*p* = 0.035, *	1.1 × ↓	300 nM	16 nM	*p <* 0.0001, ***	18.8 × ↓
NAX038	600 nM	500 nM	*p <* 0.0001, ***	1.2 × ↓	380 nM	300 nM	*p* = 0.004, **	1.3 × ↓
NAX042	380 nM	300 nM	*p* = 0.005, *	1.3 × ↓	300 nM	13 nM	*p <* 0.0001, ***	23 × ↓
NAX053	300 nM	200 nM	*p* = 0.0002, **	1.5 × ↓	200 nM	2 nM	*p <* 0.0001, ***	100 × ↓
NAX054	>2000 nM	>2000 nM	*p* = 1, NS	1 ×	>2000 nM	1000 nM	*p <* 0.0001, ***	>2 × ↓
NAX060	800 nM	400 nM	*p <* 0.0001, ***	2 × ↓	220 nM	2 nM	*p <* 0.0001, ***	110 × ↓
NAX075	>2000 nM	500 nM	*p <* 0.0001, ***	>4 × ↓	1000 nM	2.8 nM	*p <* 0.0001, ***	357 × ↓
NAX077	>2000 nM	800 nM	*p <* 0.0001, ***	>2.5 × ↓	1900 nM	1100 nM	*p* = 0.0052, *	1.7 × ↓
NAX111	300 nM	100 nM	*p <* 0.0001, ***	3 × ↓	250 nM	200 nM	*p <* 0.0001, ***	1.3 × ↓

^1^ Determined as described in [78,79,82,83,84]. *** = *p* < 0.0001, ** = *p* < 0.005, * = *p* < 0.05, and NS = not statistically significant.

**Table 2:** Effects of WT-TP53 on the sensitivity of PANC-28 PDAC cells to APR-246 ^1^.

Berberine or NAX Compound	PANC-28 + pLXSN (-APR-246)	PANC-28 + pLXSN (+12.5 nM APR-246)	*p* Value and Statistical Significance Symbol	Fold Change +/−APR-246	PANC-28 + WT-TP53 (-APR-246)	PANC-28 + WT-TP53 (-APR-246)	*p* Value and Statistical Significance Symbol	Fold Change +/− APR-246
Berberine	1600 nM	1500 nM	*p* = 0.119, NS	1.1 × ↓	1000 nM	18 nM	*p <* 0.0001, ***	55.6 × ↓
NAX012	2000 nM	500 nM	*p* < 0.0001, ***	4 × ↓	800 nM	5.5 nM	*p <* 0.0001, ***	145 × ↓
NAX014	1200 nM	700 nM	*p =* 0.0002, **	1.7 × ↓	500 nM	2 nM	*p <* 0.0001, ***	250 × ↓
NAX035	700 nM	700 nM	*p* = 1, NS	1 ×	50 nM	4 nM	*p <* 0.0001, ***	12.5 × ↓
NAX038	2000 nM	1000 nM	*p* < 0.0001, ***	2 × ↓	120 nM	2.2 nM	*p <* 0.0001, ***	54.5 × ↓
NAX042	1500 nM	1000 nM	*p* < 0.0001, ***	1.5 × ↓	32 nM	7 nM	*p <* 0.0001, ***	4.6 × ↓
NAX053	600 nM	600 nM	*p* = 1, NS	1 ×	65 nM	4 nM	*p <* 0.0001, ***	16.3 × ↓
NAX054	>2000 nM	>2000 nM	*p* = 1, NS	1 ×	>2000 nM	>2000 nM	*p* = 1, NS	1 ×
NAX060	450 nM	200 nM	*p* < 0.0001, ***	2.3 × ↓	120 nM	9 nM	*p <* 0.0001, ***	13.3 × ↓
NAX075	1200 nM	1200 nM	*p* = 1, NS	1 ×	1000 nM	45 nM	*p <* 0.0001, ***	22.2 × ↓
NAX077	1700 nM	1700 nM	*p* = 1, NS	1 ×	1500 nM	1.5 nM	*p <* 0.0001, ***	1000 × ↓
NAX111	1000 nM	1000 nM	*p* = 1, NS	1 ×	150 nM	6 nM	*p <* 0.0001, ***	25 × ↓

^1^ Determined as described in [78,82,83,84]. *** = *p* < 0.0001, ** = *p* < 0.005, and NS = not statistically significant.

## Data Availability

The data presented in this study are available on request from the corresponding authors.
